# Evaluation of Chitosan and Cellulosic Polymers as Binding Adsorbent Materials to Prevent Aflatoxin B1, Fumonisin B1, Ochratoxin, Trichothecene, Deoxynivalenol, and Zearalenone Mycotoxicoses Through an In Vitro Gastrointestinal Model for Poultry

**DOI:** 10.3390/polym9100529

**Published:** 2017-10-19

**Authors:** Bruno Solís-Cruz, Daniel Hernández-Patlán, Eric Beyssac, Juan D. Latorre, Xochitl Hernandez-Velasco, Ruben Merino-Guzman, Guillermo Tellez, Raquel López-Arellano

**Affiliations:** 1 Unidad de Investigación Multidisciplinaria. Facultad de Estudios Superiores Cuautitlán, Universidad Nacional Autónoma de Mexico, Cuautitlán Izcalli, Estado de Mexico 54714, Mexico; bruno_sc@comunidad.unam.mx (B.S.-C.); danielpatlan@comunidad.unam.mx (D.H.-P.); rlajjd@yahoo.com.mx (R.L.-A.); 2 Clermont-Université, Université d’Auvergne, EA4678, Conception Ingénierie et Développement de L’aliment et du Médicament, 63001 Clermont-Ferrand, France; eric.beyssac@udamail.fr; 3 Department of Poultry Science, University of Arkansas, Fayetteville, AR 72701, USA; juandlatorre@gmail.com; 4 Departamento de Medicina y Zootecnia de Aves, Facultad de Medicina Veterinaria y Zootecnia, Universidad Nacional Autónoma de México, Ciudad de Mexico 04510, Mexico; xochitl_h@yahoo.com (X.H.-V.); onirem@unam.mx (R.M.-G.)

**Keywords:** adsorption, cellulosic polymers, chitosan, in vitro, mycotoxins

## Abstract

Mycotoxins are secondary toxic metabolites that are produced by fungi representing threats to human and animal health. The objective of this study was to evaluate the adsorption capacity of Chitosan (CHI), and three cellulosic polymers (HPMC, CMC, and MCC), on six mycotoxins (AFB_1_; FUB_1_; OTA; T-2; DON; and, ZEA) using an in vitro digestive model for poultry. The adsorbent capacity of the materials in the supernatant of each compartment was evaluated by a non-competitive chemiluminescent assay. Control groups with no adsorbent material had an adsorption value of 0.00% against all six mycotoxins that were evaluated. All four materials tested showed significant (*p* < 0.05) binding activity against all of the mycotoxins when compared with the control non-treated group. However HPMC, CMC, and MCC showed better adsorbent capacity when compared with CHI.

## 1. Introduction

Mycotoxins are secondary toxic metabolites produced by filamentous fungi which, even at low concentrations, represent an important danger for both animal and human health [1,2]. Currently, over 300 mycotoxins have been identified worldwide, being aflatoxins, ochratoxins, zearalenone, trichothecenes, and fumonisins, the most frequently found with synergistic toxic effects reported when more than one of these mycotoxins are present in the feed [3,4]. Mycotoxins are chemically and structurally different, representing serious public health risk factors since mycotoxins have been shown to have carcinogenic, teratogenic, nephrotoxic, and hepatotoxic effects after the consumption of contaminated grains or animal food products [5,6,7]. On the other hand, mycotoxins are equally important in the animal food industry, causing significant economic losses due to diminished performance and productivity, decreased reproductive parameters, and an increased mortality rate associated with the toxicological effects in liver, kidneys, and immune system [1,6,8]. Researchers have developed some methods in order to reduce the harmful effects of grains contaminated with mycotoxins. These include physical (thermal and irradiation inactivation); chemical (ozonation and ammoniation); and, biological (bacterial degradation or adsorption) [6,9,10,11]. Nevertheless, toxin sequestering agents are the most common and reliable products used for the feed industry due to its economic practicality and aptness for nutritional insight [12,13,14]. Several studies have demonstrated that cellulosic materials have adsorption capacities for heavy metal ions and other pollutants [15,16,17]. Similarly, some researchers have evaluated the binding activity of chitosan against several mycotoxins [18,19,20,21,22]. On the other hand, some in vitro methods have been developed to evaluate the adsorbent capacity of mycotoxin sequestering products [23,24,25,26]. However, these methods may not be directly applicable to poultry diets because they do not use the successive incubation at different pH and enzyme activity conditions similar to the different gastrointestinal compartments of poultry. Hence, the objective of this study was to evaluate and compare the adsorption capacity of chitosan and three cellulosic polymers on Aflatoxin B1 (AFB_1_); Fumonisin B1 (FUB_1_); Ochratoxin (OTA); Trichothecene (T-2); Deoxynivalenol (DON); and, Zearalenone (ZEA), using an in vitro digestive model that simulates three gastrointestinal compartment of poultry.

## 2. Materials and Methods

### 2.1. Mycotoxins and Adsorbents

Mycotoxins AFB_1_, FUB_1_, OTA, T-2, DON, and ZEA were obtained from Cayman Chemical Company (Ann Arbor, MI, USA). In this study, deacetylated 95%, high-molecular-weight (350 kDa) Chitosan (CHI, Paragon Specialty Products, LLC, Rainsville, AL, USA), and three cellulosic polymers, Hydroxypropyl methylcellulose (HPMC, Methocel™, Chempoint, Bellevue, WA, USA), Sodium Carboxymethylcellulose (CMC, Aqualon™, Ashland, Columbus, OH, USA), and Microcrystalline Cellulose (MCC, Avicel™, FMC, Philadelphia, PA, USA) were used to evaluate and compare their adsorbent capacity against mycotoxins. A chitosan solution was prepared by weighing 1 g of food grade chitosan and dissolving it in 100 mL of acetic acid 1% (*v*/*v*) aqueous solution. Then, this solution was dropped into 100 mL of NaOH 0.5 M solution and stirred for 40 min at 600 rpm. The formed chitosan particles were rinsed three times with pure water and dried at 40 °C for 12 h. The resulting products were ground and sieved to collect the particles.

### 2.2. Mycotoxin Solutions

Solutions were prepared by dissolving each mycotoxin separately. Primary standard solutions of AFB_1_, FUB_1_, OTA, T-2, DON (100 μg/mL), and ZEA (500 μg/mL) were prepared in dimethyl sulfoxide (DMSO). These solutions were then diluted to 4, 30, 8, 8, 30, and 30 μg/mL, respectively, using distilled water. Next, the AFB_1_, OTA and T-2 solutions (4, 8 and 8 μg/mL) were diluted to 0.32, 0.64, and 1.92 μg/mL using distilled water. The final concentrations were AFB_1_ (0.32 μg/mL), FUB_1_ (30 μg/mL), OTA (0.64 μg/mL), T-2 (1.92 μg/mL), DON (30 μg/mL), and ZEA (30 μg/mL).

### 2.3. Inoculation of Feed Material

The experimental diet was formulated to approximate the nutritional requirements of broiler chickens, as recommended by the National Research Council [27], and adjusted to breeder’s recommendations [28]. No antibiotics were added to the feed (Table 1). For spiking, 3.0 g of blank feed material were fortified separately with 0.5 mL of mycotoxin spiking solution in order to reach the following mycotoxin concentrations in the feed: AFB_1_ (50 μg/kg), FUB_1_ (5000 μg/kg), OTA (100 μg/kg), T-2 (300 μg/kg), DON (5000 μg/kg), and ZEA (5000 μg/kg). Then, the spiked feed was incubated overnight in the dark at 40 °C in order to evaporate to dryness. The samples were prepared as independent triplicates for each experiment.

### 2.4. In Vitro Digestive Model

In vitro studies for the assessment of the efficiency of polymers in adsorbing mycotoxins were subjected to a simulated gastrointestinal poultry model. All of the steps of this model were performed by quintuplicate at 40 °C to simulate poultry body temperature according to previous publications with minor modifications [29,30]. Briefly, for all of the gastrointestinal compartments simulated during the in vitro digestion model, a BOD incubator (Biochemical oxygen demand incubator, model 2020, VWR, Houston, TX, USA) customized with an orbital shaker (Standard orbital shaker, model 3500, VWR, Houston, TX, USA) was used for mixing the feed content in the experimental tubes at 19 rpm. Additionally, all of the tube samples were held in a 30 degrees inclination position to facilitate the proper blending of feed particles and the enzyme solutions incorporated throughout the assay. The first gastrointestinal compartment simulated was the crop. Three grams of contaminated feed with each mycotoxin and 50 mg of each of the binding products respectively, were mix with 10 mL of 0.03 M hydrochloric acid (HCl, catalog no. HX0607-2, EMD Millipore corporation, Billerica, MA, USA) in 50 mL polypropylene centrifuge tubes. Each tube was mixed vigorously, reaching a pH value around 5.2. Then, all of tubes were incubated for 30 min. The second gastrointestinal compartment simulated was the proventriculus, where 3000 U of pepsin (catalog no. P700, Sigma-Aldrich, St. Louis, MO, USA) per g of feed were used and 2.5 mL of 1.5 M HCl were added to each tube, reaching a pH between 1.4 to 2.0, at that time all of the tubes were incubated for 45 min. The third and final gastrointestinal compartment simulated was the intestinal section. In this case, 6.84 mg of 8 x pancreatin (catalog no. P7545, Sigma-Aldrich, St. Louis, MO, USA) were used per g of feed and included in 6.5 mL of 1.0 M sodium bicarbonate (NaHCO_3_, catalog no. S6014, Sigma-Aldrich, St. Louis, MO, USA), the pH ranged between 6.4 and 6.8, and all tube samples were incubated for 2 h. The complete in vitro digestion process took 3 h and 15 min. After incubation, all of the tubes were centrifuged at 2000 ×*g* for 30 min, to separate solids and supernatant, which was filtered, collected, and stored at −20 °C until analysis of residual unbound mycotoxin. At the same time, blank controls were prepared without the addition of adsorbent polymers. The mycotoxin values obtained for the blank controls without adsorbents were used as a reference.

### 2.5. Quantitation of the Percentage of Adsorbed Mycotoxins

The adsorption percentage of mycotoxin for each polymer was calculated as follow:

Adsorption (%) = (C_i_ − C_s_)/Ci × 100, where C_i_ is the mycotoxin concentration in blank control (ng/mL); C_s_ the amount of mycotoxin in the supernatant (ng/mL).

### 2.6. Analysis and Quantification of Mycotoxins

Unbound mycotoxins analysis was carried out using the Myco 7 biochip array kit (EV4065A; Randox Food Diagnostics, Crumlin, UK). The kit contains multianalyte biochips, assay diluent, conjugate diluent, multianalyte conjugate, a set of multianalyte calibrators (spanning the range of each assay), multianalyte controls, signal reagent, washing buffer, calibration disc, and barcodes. The biochips were supplied in carriers (3 × 3 biochips per carrier), and a carrier handling tray was provided with the system that allows the simultaneous handling of six carriers (54 biochips). Data were generated and processed with the semiautomated benchtop biochip analyzer Evidence Investigator (EV3602; Randox Food Diagnostics, Crumlin, UK), this is a non-competitive chemiluminescent assay using a unique image processing software to translate the light signal generated from the chemiluminescent reactions into an analyte concentration.

### 2.7. Statistical Analysis

STATGRAPHICS Centurion XV software computer was used to conduct statistical analysis (Statistical Graphics Co., Rockville, MD, USA. 2007). A multi-factor ANOVA was used to construct a statistical model describing the impact of the used polymer with each mycotoxin on the percentage of adsorption. Furthermore, a multiple sample comparison test was designed to determine if there were or not significant differences in the percentage of adsorption of mycotoxins on each polymer. To determine which polymers were significantly different from others, a multiple range test was performed, where the method used to construct the intervals and to discriminate among the means was Fisher’s least significant difference test (LSD), with a 95% of confidence associated with each interval.

## 3. Results

**Figure 1:**
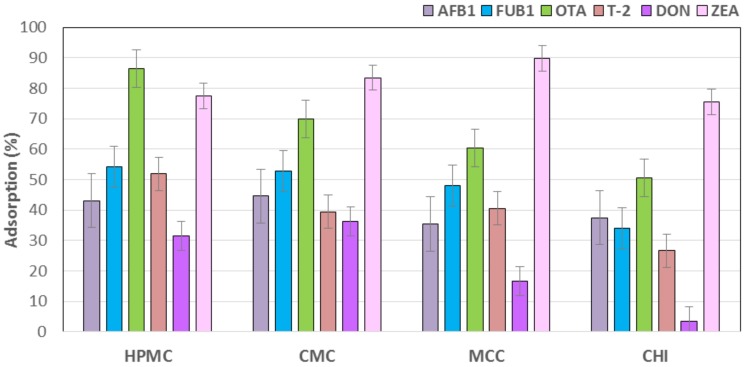
Percentage of adsorption of multiple mycotoxins on several adsorbents. Bars are the mean values. Error bars displays an interval around each mean, which are based on Fisher’s least significant difference (LSD) procedure.

The results of the evaluation of adsorbents materials for mycotoxins in an in vitro gastrointestinal model are summarized in Table 2 and Figure 1. In Table 2, control groups with no adsorbent material had an adsorption value of 0.00%, denoting the lack of adsorbent activity against all six mycotoxins evaluated. For AFB_1_ the best adsorbent materials were CMC (44.6%) and HPMC (43.1%), followed by CHI (37.5%) and MCC (35.4%), respectively (*p* < 0.05). For FUB_1_ the materials that showed the highest significant adsorbent capacity were the cellulosic polymers, HPMC (54.1%); CMC (52.9%) and MCC (48.1%); and, followed by the CHI (34%). Interestingly, HPMC was the material that showed the best binding activity against OTA with 86.3%, followed by CMC (69.8%) and MCC (60.5%). The material with the lowest adsorbent capacity against this mycotoxin was CHI (50.6%). The best sequestering agents against T-2 were, once again, the cellulosic materials HPMC (51.9%); MCC (40.6%); and, CMC (39.5%). CHI (26.7%) showed moderate adsorbent capacity against T-2. For DON, CMC (36.3%), and HPMC (31.4%) were the two materials that showed the best binding activity, they were followed by MCC (16.7%). No significant differences were observed with CHI (3.5%) when compared with control group (0.0%). MCC (89.7%) and CMC (83.5%) showed the highest sequestering activity against ZEA. They were followed by HPMC (77.6%) and CHI (75.6%) (Table 2; Figure 1).

## 4. Discussion

Mycotoxins are compounds produced by fungi that diverge in their chemistry and biological effects [5]. It is uncommon to find grains that are contaminated with a single mycotoxin, due to the fact that many fungal species can grow and synthetize mycotoxins under similar environmental conditions. Moreover, animal diets are made up of several sources, which may be contaminated with a different mycotoxin or more than one mycotoxin. Therefore, the most practical dietary approach to prevent mycotoxicosis in animals is the use of adsorbents [31,32,33,34]. However, single mycotoxin adsorbents lack binding effects against multiple mycotoxins [35]. Several inorganic materials, such as bentonites and aluminosilicates, or clays in general, have been shown to have adsorptive properties to reduce the toxic effect of aflatoxins, but they have limited efficacy against other mycotoxins [33,35,36]. Additionally, they need to be incorporated at high levels affecting the absorption of some dietary nutrients, hence reducing the performance of the animals. On the other hand, some of these compounds also contain heavy metals, limiting their use as feed additives and impede the use of the manure from treated animals as fertilizer. Therefore, several studies have been carried out on other types of binders during the last decade [18,26]. Some of these materials offer a great potential to adsorb mycotoxins in feed, however, a material does not exist yet that meets all of the desirable features. Natural or synthetic polymers are large molecules that are composed of many monomers that have extensive properties. They range from familiar synthetic plastics to natural biopolymers, such as cellulose, the most abundant biopolymer and source of carbon on earth [37]. Their large molecular mass relative to a small molecule produces unique physical properties playing important roles in our society. In the present study, four adsorbent materials were tested, including biopolymer chitosan (CHI) and three cellulosic polymers (HPMC, CMC, and MCC). This previously published model [30] has the advantage that includes all of the pH changes through the digestive tract of poultry (5.2 for the crop; 1.8 for the proventriculus; and 6.6 for the intestinal tract). All materials tested have extensive use as excipients in the pharmaceutical, and food industries [37,38,39]. In particular, the three cellulosic materials HPMC, CMC, and MCC are used as adsorbents for the removal of dyes from aqueous media [40,41]. As far as we know, this is the first time these materials have been tested for their binding activity as adsorbent against mycotoxins. Yet, it was remarkable to observe that these materials showed excellent sequestering activity against all six mycotoxins evaluated. HPMC had an outstanding binding activity, however, HPMC is a nonionic polymer, which in contact with water or gastrointestinal fluids swell and make a gel layer around the dry core of the polymer matrix [42]. This gel layer formation leads to an increase in viscosity, which disqualifies it as a good candidate as adsorbent material in the animal industry, particularly poultry. CMC and MCC had a similar binding activity against all six mycotoxins, but CMC (36.2%) showed a significant sequestering activity against DON when compared with MCC (16.7%). CMC is an anionic polysaccharide whose monomer units contain three OH groups and these groups are available for hydrogen bonding. Studies have shown that the main forces responsible for CMC adsorption capacity is a combination of electrostatic interactions and hydrogen bonding [43]. CMC is neutral at low pH values but hydrolyzes at high pH values (at high pH, CMC loses its positive Na^+^ counter-ion, acquiring a negative charge) [44] suggesting that it will behave well under different pH conditions of the gastrointestinal tract of poultry and as a mycotoxin adsorbent. On the other hand, MCC is a purified and partially depolymerized cellulose, which presents considerable chemical and physical inertness. The adsorption mechanisms of MCC correspond to a monolayer adsorption at its surface. Some studies have shown that electrostatic attraction (physical adsorption) is the dominant adsorption mechanism of MCC [17]. The results of the present study propose the potential of MCC as a mycotoxins adsorbent, although further improvement is still required to increase the adsorption capacity. With respect to the biopolymer, CHI has been shown to have promising uses as an adsorbent for the removal of various mycotoxins, heavy metal ions, and dyes [18]. Furthermore, it has been tested in the removal of OTA from contaminated drinks, demonstrating that CHI can reduce the levels of this mycotoxin [19,45]. In the present study, CHI showed a moderate adsorbent capacity against five of the six mycotoxins evaluated when compared with the cellulosic polymers, even though, it did not show good adsorption capacity against DON. We tested the effectiveness of non-cross-linked chitosan, however the formation of crosslinked chitosan particles could improve its adsorption capability. The results of this in vitro study suggest that HPMC, CMC, and MCC were the materials that showed the best adsorbent capacity against all six mycotoxins evaluated when compared with CHI. However, the increase in viscosity caused by HPMC, disqualifies it as a good candidate as adsorbent material for monogastrics animals. On the other hand, CMC and MCC also demonstrated remarkable binding properties against all six of the mycotoxins assessed, suggesting that they can be candidates for poultry and other monogastic animals.

## 5. Conclusions

The results of the present study, suggest that cellulosic polymers have the highest adsorption capability for all of the mycotoxins. Although high-molecular-weight and non-cross-linked CHI showed significant adsorbent properties against five of the six mycotoxins that were evaluated, it is possible that chitosan of different molecular weight, degree of deacetylation, or cross-linked might show different adsorptive properties against these mycotoxins. In summary, in the present study, the lowest adsorbent capacity by all four materials was for DON, and the highest mycotoxin binding activities were observed against ZEA and OTA.

## Figures and Tables

**Table 1:** Ingredients (%). Diet based on corn-soybean for broiler chickens.

Item	Diet based on corn-soybean
Ingredients	
Corn	54.64
Soybean meal	36.94
Vegetable oil	3.32
Dicalcium phosphate	1.58
Calcium carbonate	1.44
Salt	0.35
DL-Methionine	0.25
Vitamin premix ^1^	0.30
L-Lysine HCl	0.10
Choline chloride 60%	0.10
Mineral premix ^2^	0.30
Antioxidant ^3^	0.15

^1^ Vitamin premix supplied the following per kg: vitamin A, 20,000,000 IU; vitamin D3, 6,000,000 IU; vitamin E, 75,000 IU; vitamin K3, 9 g; thiamine, 3 g; riboflavin, 8 g; pantothenic acid, 18 g; niacin, 60 g; pyridoxine, 5 g; folic acid, 2 g; biotin, 0.2 g; cyanocobalamin, 16 mg; and ascorbic acid, 200 g (Nutra Blend LLC, Neosho, MO 64850). ^2^ Mineral premix supplied the following per kg: manganese, 120 g; zinc, 100 g; iron, 120 g; copper, 10–15 g; iodine, 0.7 g; selenium, 0.4 g; and cobalt, 0.2 g (Nutra Blend LLC, Neosho, MO 64850).^3^ Ethoxyquin.

**Table 2:** Evaluation of adsorbent materials for mycotoxins in an in vitro gastrointestinal model. ^1^

**Adsorbent**	**Mycotoxin**								
	**AFB_1_ (ng/mL)**		**Adsorption ^2^ (%)**	**FUB_1_ (ng/mL)**		**Adsorption ^2^ (%)**	**OTA (ng/mL)**		**Adsorption ^2^ (%)**
	**Initial**	**Unbound**		**Initial**	**Unbound**		**Initial**	**Unbound**	
Control	12.00	12.00 ± 0.43 ^a^	0.00 ^b^	1832.33	1832.33 ± 43.21 ^a^	0.00 ^c^	28.32	28.32 ± 2.52 ^a^	0.00 ^d^
HPMC		6.833 ± 1.12 ^b^	43.06 ± 9.33 ^a^		840.67 ± 67.98 ^c^	54.12 ± 3.71 ^a^		3.87 ± 0.48 ^d^	86.35 ± 1.70 ^a^
CMC		6.65 ± 0.85 ^b^	44.58 ± 7.12 ^a^		863.00 ± 54.51 ^c^	52.90 ± 2.97 ^a^		8.55 ± 0.33 ^c,d^	69.81 ± 1.15 ^b^
MCC		7.75 ± 0.45 ^b^	35.42 ± 3.78 ^a^		951.00 ± 44.75 ^c^	48.10 ± 2.44 ^a^		11.18 ± 2.24 ^b,c^	60.51 ± 7.90 ^b,c^
CHI		7.50 ± 0.31 ^b^	37.50 ± 2.55 ^a^		1208.67 ± 144.15 ^b^	34.04 ± 7.87 ^b^		13.98 ± 0.90 ^b^	50.63 ± 3.16 ^c^
SEM ^3^	-	0.70	5.63	-	80.30	4.25	-	1.58	3.92
*p*-value	-	0.0017	0.0013	-	0.0000	0.0000	-	0.0000	0.0000
**Adsorbent**	**Mycotoxin**								
	**T-2 (ng/mL)**		**Adsorption ^2^ (%)**	**DON (ng/mL)**		**Adsorption ^2^ (%)**	**ZEA (ng/mL)**		**Adsorption ^2^ (%)**
	**Initial**	**Unbound**		**Initial**	**Unbound**		**Initial**	**Unbound**	
Control	174.83	174.83 ± 7.20 ^a^	0.00 ^d^	99.35	99.35 ± 1.86 ^a^	0.00 ^c^	115.33	115.33 ± 7.17 ^a^	0.00 ^c^
HPMC		84.17 ± 6.27 ^c^	51.86 ± 3.59 ^a^		68.12 ± 4.09 ^c^	31.43 ± 4.12 ^a^		25.83 ± 2.83 ^b^	77.60 ± 2.46 ^b^
CMC		105.83 ± 4.60 ^c^	39.47 ± 2.63 ^b^		63.31 ± 3.92 ^c^	36.27 ± 3.94 ^a^		19.00 ± 5.25 ^b,c^	83.53 ± 4.55 ^a,b^
MCC		103.83 ± 3.98 ^c^	40.61 ± 2.28 ^b^		82.77 ± 2.96 ^b^	16.69 ± 2.98 ^b^		11.83 ± 3.06 ^c^	89.74 ± 2.65 ^a^
CHI		128.17 ± 10.54 ^b^	26.69 ± 6.03 ^c^		95.82 ± 1.92 ^a^	3.55 ± 1.93 ^c^		28.17 ± 1.20 ^b^	75.58 ± 1.04 ^b^
SEM ^3^	-	6.92	3.50	-	3.10	3.00	-	4.42	2.64
*p*-value	-	0.0000	0.0000	-	0.0000	0.0000	-	0.0000	0.0000

^1^ Each value represents the mean ± standard error. ^2^ Calculated in comparison to the control treatment containing no sequestering agent. ^a–d^ Values labeled with the same superscript in a column are not significantly (*p* < 0.05). ^3^ Standard error of the means.
